# Transformative Experiences, Cognitive Modelling and Affective Forecasting

**DOI:** 10.1007/s10670-022-00523-z

**Published:** 2022-03-02

**Authors:** Marvin Mathony, Michael Messerli

**Affiliations:** 1 Humboldt-Universität zu Berlin, Berlin School of Mind and Brain, Berlin, Germany; 2 Institute of Philosophy, Zollikerstrasse 117, 8008 Zürich, Switzerland

## Abstract

In the last seven years, philosophers have discussed the topic of transformative experiences. In this paper, we contribute to a crucial issue that is currently under-researched: transformative experiences' influence on cognitive modelling. We argue that cognitive modelling can be operationalized as affective forecasting, and we compare transformative and non-transformative experiences with respect to the ability of affective forecasting. Our finding is that decision-makers’ performance in cognitively modelling transformative experiences does not systematically differ from decision-makers’ performance in cognitively modelling non-transformative experiences. This claim stands in strict opposition to L.A. Paul’s main argument.

## Introduction

L.A. Paul’s seminal book, *Transformative Experience* (2014), has proven immensely influential in both academic and non-academic discussions. Central to the philosophical research is the concept of transformative experience, that is, an experience which is both *epistemically* and *personally* transformative. An epistemically transformative experience teaches you something that you could not have learned without having that specific type of experience. Having the experience of being a parent, for instance, may be the only way to know what it is like to be a parent. An experience that is also personally transformative changes one’s core preferences. Being a mother or a father can change some of one’s most fundamental preferences. Thus, being a parent is a transformative experience, and the choice of whether to have a child is a transformative decision.

Paul argues that there is a certain way one goes about making transformative decisions. The most important part of the deliberation concerns what one’s future life will be like (Paul, 2014, p. 3), and to get an idea of that, we use *cognitive modelling*. Cognitive modelling, a crucial element in Paul’s argument, can be understood as a capacity to estimate the desirability of alternative states of affairs; we mentally simulate the alternatives in order to assess their value. As an illustration of this capacity, suppose you are considering whether to become a parent or stay childless. In order to assign a value to the alternatives, *having a child* and *not having child*, you create a kind of mental cinema. In the first movie, you do have a child; in the second, you live a childless life. Importantly, you not only imagine what you will do (and will not do) but also how all of this will feel to you.

Paul argues that this kind of simulation fails in the case of transformative experiences. The main claim of her book is that “the epistemically and personally transformative nature of transformative experience creates problems for an individual-level decision procedure based on cognitive modelling” (Paul, 2014, p. 124). The lack of research on this point is surprising, given its place in Paul’s work. There has been almost no research on transformative experiences and cognitive modelling, although there are some exceptions. Fairly recently, some scholars have started to investigate certain aspects of Paul’s notion of cognitive modelling. This work includes a critique on (implausible) discontinuities in the evaluation of outcomes—the so-called *shark problem* (Campbell & Mosquera, 2020)—and criticism concerning the prevalence of the phenomenon (Bykvist and Stefánsson, 2017). However, the existing research does not address Paul's main claim that the problem of cognitive modelling arises in virtue of transformative experiences.

We have therefore taken on the task of providing the first meta-analysis on whether cognitive modelling fails because of the transformative nature of the transformative decision. In order to do that, we operationalize cognitive modelling as affective forecasting and compare transformative and non-transformative experiences with respect to the ability of affective forecasting. The result of our meta-analysis is that transformative experiences are no more difficult to model cognitively than other experiences.We will proceed as follows. In Sect. 2, we reconstruct Paul’s view on cognitive modelling in greater detail. Section 3 introduces our argument. In Sect. 4, we argue that the ability to cognitively model can be empirically tested with the method of affective forecasting. Section 5 presents the results of the meta-analysis; in particular, people’s predictions of their future levels of happiness are inaccurate independently of whether the experience is transformative. In Sect. 6, we cover potential objections to our approach and findings. The details of the meta-analysis are in the appendix.

## Paul’s View on Cognitive Modelling

There are different ways to reconstruct Paul's challenge for decision-making as well as her rationale for assigning cognitive modelling as the centrepiece of her argument.^1^ We think that cognitive modelling stands at the centre of Paul’s argument because of its connection with authenticity. Paul advocates a very distinct view of authenticity. *Paul-Authenticity* means “authentic self-governance informed by knowledge via experiential or imaginative acquaintance with objects of deliberation” (Paul’s characterization of authenticity in her *Teaching Guide to Transformative Experiences*: p. 9). Moreover, the so-called *subjective value*—i.e., the value of experiencing a certain outcome of a decision, such as the value of what it is like to be a parent—is crucial for Paul in this regard. She writes:

In my view, for many big, life-changing decisions, you want to authentically assess your options by assessing the subjective value of your possible future lived experiences. Ideally, the assessment involves a determination of the subjective value of each possible outcome of your decision, that is, each possible lived experience, by imaginatively grasping what it would be like for you to live in that future. (Paul, 2015b, p. 807)

Simply put, in Paul’s view, an authentic choice involves cognitive modelling and an agent assigning subjective value after cognitively modelling each outcome (there might be exceptions where one has sufficient evidence of subjective value prior to modelling).

Paul provides a clear account of how she thinks cognitive modelling works. One runs a cognitive simulation to project oneself into experiencing each possible outcome of the decision at hand. This is to say that, for each outcome, one puts oneself in the shoes, i.e. the first-person perspective, of a future self that is materialized in that outcome. That way, one figures out *what it would be like* for oneself to have such an experience. Based on what that would be like, one assigns a subjective value to each hypothetical outcome. The goal is to identify the action that leads to the outcome with the highest expected value—to figure out which option *should* be taken.

Thus, Paul's approach involves a descriptive and a normative part. The normative part states that one *should* choose the alternative with the maximum expected value (Paul, 2015a, p. 3; Reuter & Messerli, 2018, p. 317). The descriptive part of *authentic decision-making* concerns the way in which subjective values are assigned to outcomes.

Importantly, Paul argues that the ability to cognitively model is limited when confronted with a transformative choice. Epistemically transformative experiences are problematic because they cannot be imagined without having lived through them. Personally transformative experiences are problematic because they change one’s core preferences. This means that even if one could assign values to the outcomes before undergoing a transformative experience (which one cannot, in Paul’s view), these values could be different from the values one would assign to the outcomes after having gone through the experience.

Transformative experiences thereby pose two distinct problems for ranking alternatives based on subjective values assigned after cognitive modelling. First, one needs to find out what a relevant experience would be like. Second, one needs to figure out how much one values the what-it-is-like character of the experience. The first ability is limited when the decision-maker has never had the experience before—given that the experience in question is epistemically transformative. The second ability is limited when the experience would inevitably change one’s core preferences, so that one does not know how a future self would value certain outcomes—in the case of a personally transformative experience. In both cases, no subjective value can be assigned to outcomes. Thus, the ranking of options cannot be determined authentically.^2^

## The Structure of our Argument

Having briefly portrayed Paul’s view on cognitive modelling in the last section, we will now present the structure of our argument.^3^ Recall that Paul’s main point is that cognitive modelling fails because of the transformative nature of the decision. According to our finding, however, it is false that cognitive modelling fails because of the transformative nature of the decision.

The general version of our argument has the following form:

(A) If the transformative nature creates problems for cognitive modelling, then these problems lead to some measurable effect (if *a* then *b*).
(B) There is no measurable effect (*not b*).

C: It is *not* the case that the transformative nature of transformative experiences creates problems with cognitive modelling (*Modus Tollens* from (A) and (B)).

Note that we assume that transformative and non-transformative experiences do not differ systematically from each other except for the transformative nature.^4^ The more particular version of our argument, in which the crucial operationalization and result of our meta-analysis are taken into account, works as follows:

(i) If the transformative nature creates some problems for affective forecasting, then these problems lead to some measurable effect on the accuracy of predictions in affective forecasting surveys (if *a* then *b*).
(ii) Affective forecasts concerning transformative and non-transformative experiences are equally accurate (*not b*).

C: It is *not* the case that the transformative nature of transformative experiences creates problems with cognitive modelling (*Modus Tollens* from (i) and (ii)).

*If* one accepts that cognitive modelling can be operationalized as affective forecasting, i.e. that problems with cognitive modelling manifest in the accuracy of predictions in affective forecasting surveys, it follows that it is not the transformative nature of transformative experiences that creates the problems with cognitive modelling. The main reason is that people are not worse in predicting their future happiness in the context of transformative experiences (premise (ii), the result of our meta-analysis). Otherwise stated, if affective forecasts concerning transformative and non-transformative experiences are equally accurate (premise (ii)), the transformative nature cannot be the source of the problems for cognitive modelling. For if that were the case, then, according to premise (i), it would have a measurable impact on the affective forecasting performance. As our argument stands and falls with the operationalization of cognitive modelling as affective forecasting, we demonstrate its validity in the next section.

## Cognitive Modelling and Affective Forecasting

First, we introduce affective forecasting and its connection to happiness (4.1). Second, we explain why affective forecasting is a promising operationalization of cognitive modelling (4.2). Third, we argue that subjective values can be operationalized as measures of happiness (4.3).^5^

### Affective Forecasting and Happiness

Affective forecasting can be understood as our capacity to predict how we will feel in the future. More specifically, affective forecasting is our ability to predict the impact that certain experiences will have on our happiness. When deliberating about the future, people often take into consideration their future level of happiness. For example, if one considers having a child, one is not only concerned with parenthood’s consistency with personal goals, such as career plans, and external factors, like financial cost. One also considers how it will feel to be a parent (see Reuter & Messerli, 2018, for an empirical investigation into the relative importance of the various criteria). Importantly, if one wants to know how it will feel to experience *x* (e.g. being a parent), it is plausible that one will try to *imagine* how *x* will feel. The vast literature on affective forecasting is dedicated to measuring this ability.

In order to understand why affective forecasting measures our ability to predict the impact of future experiences on our happiness, one has to understand the basic methodology behind affective forecasting studies. Most of these studies use self-report scales. Normally, there are two groups of participants: ‘forecasters’ and ‘experiencers’. Before the so-called *focal event* (e.g. an HIV-positive diagnosis or a football game) takes place, all participants answer a question about their baseline happiness. Then, forecasters receive a questionnaire asking them to predict how happy they will be after the focal event. After the focal event has taken place, experiencers receive a questionnaire asking them how happy they are in general or how they felt about the event. Finally, psychologists compare the values of forecasters’ expected happiness and the experiencers’ reported actual happiness. These values should not differ from each other if we are able to perfectly imagine how we will feel in the future. The reality, however, is that these values do differ. Forecasters systematically overpredict the impact that the focal event will have on their future level of happiness. This gap between predicted value and experienced value is sometimes termed ‘misprediction’ (see e.g. Gilbert & Wilson, 2000). For example, the prediction that having children will make you happier than you have ever been might not correspond to the actual net effect of having a child on your happiness.

Numerous studies have found that people are not very good at predicting the *intensity* and *duration* of their future feelings. This is known as the so-called ‘impact bias’; we incorrectly predict the emotional intensity and the emotional duration of future feelings (see e.g. Rachman & Arntz, 1991; Buehler & McFarland, 2001; Gilbert et al., 2002). When we try to predict how happy we will be, we suffer cognitive biases. These biases negatively affect our imagination such that it becomes less accurate.

The crucial point for us is that affective forecasting studies address both transformative and non-transformative experiences. For example, affective forecasting studies are focused around transformative events, such as a serious disease, or non-transformative events, such as a football game. We will discuss how to categorize a study as investigating a transformative or non-transformative experience in Sect. 5; and we will demonstrate that people’s predictions of the intensity and duration of their future happiness tend to be inaccurate, independently of whether the experience is transformative. In the following, we will explain why findings about affective forecasting have bearing on our knowledge about the ability to cognitively model.

### Why Affective Forecasting is a Promising Operationalization of Cognitive Modelling

Let us start by considering alternative operationalizations of cognitive modelling. Umbrella terms like ‘prospection’ or ‘mental time travel’ might come to mind. More specifically, a particular form of prospection, *episodic foresight,* defined as the imagination of future events (Suddendorf, 2010), might seem to be a promising candidate for operationalizing cognitive modelling. Indeed, the similarity is striking at first sight: in studies investigating episodic foresight, probands are also asked to imagine how a particular event will unfold. However, there are two main problems with operationalizing cognitive modelling as episodic foresight. First, the simulation subjects are asked to perform in episodic foresight studies is not subsequently tested for its accuracy. That is to say, in order to test whether people have problems with cognitive modelling, one has to in some way compare the simulations to the outcomes. In affective forecasting questionnaires, this is done by comparing the responses of forecasters and experiencers. Second, episodic foresight studies do not specifically investigate a simulation of how happy people will feel. Rather, the most common simulations asked for in this branch of research are phenomenological ratings such as “vividness, sensory detail, visual perspective, emotional valence and intensity” (Miloyan & McFarlane, 2018, p. 353). The point is that, in Paul’s view, for a simulation of how one will feel in the future, the question of happiness is more important than the question of, say, sensory detail or visual perspective.^6^ However, these two problems are not categorical. This is to say that if an episodic foresight task includes a measure of happiness, it can be a suitable operationalization of cognitive modelling. An appropriate paradigm would be an episodic foresight task where participants provide emotional valence ratings. If such a paradigm were to include a measure of accuracy, it could be used in future research to test the results presented in this paper. We would like to note, however, that there might be little difference between an episodic foresight study with these qualifications and an affective forecasting study.^7^

It is also crucial to emphasize that we are not alone in claiming that cognitive modelling can be operationalized as affective forecasting. Such a relation has been hinted at in the literature (Kauppinen, 2015; McCoy & Ullman, 2019), but to our knowledge it has never been followed by an analysis of the corresponding empirical data—an omission that we rectify in Sect. 5.

To sum up, the main advantage of operationalizing cognitive modelling as affective forecasting is that, as far as we are aware, no other operationalization includes the conjunction of a test of accuracy and a clear link to happiness. In affective forecasting questionnaires, people are usually asked to predict their general happiness on a one-dimensional scale (often scales of 1–7 or 1–9). Admittedly, one might doubt whether a rating on such a scale is in line with Paul's notion of subjective value.

### Subjective Value and Happiness

While we do not make claims about the nature of subjective value or the nature of happiness, we do think that, in Paul’s view, there is a tight link between the two concepts. This is perhaps clearest as she discusses the decision whether or not to become a parent:

Second, unless you know which characteristics you need to have in order to have an outcome with a high subjective value, you won’t know how to evaluate the anecdotal testimony from your friends and family. You don’t know which psychological similarities and differences between you and your friends and family are the ones that would be relevant to increases in well-being or happiness, and there isn’t any well-documented empirical work showing correlations between personalities of a particular type and increases in well-being or happiness after having a child. (Paul, 2014, p. 89)

Here Paul argues that if you would know which psychological traits caused friends and family to be happy in a relevant situation, and know that you have those traits as well, you could infer that the respective outcome would have a high subjective value for you. Thus, she makes clear that an outcome in which you would be happy has a high subjective value.

In the same chapter, Paul argues that if you want to maximize your expected subjective value without making use of any cognitive simulations, but solely using the available empirical data, you should decide not to have a child. This is because the data suggests that parents in all comparable groups have lower levels of happiness and subjective well-being (pp. 86). Hence, lower happiness or well-being corresponds to lower subjective values. This link is apparent in several other passages of Paul’s work.^8^

We grant that the connection between subjective value and happiness is a sensitive point—not least because the notion of ‘subjective value’ has not yet been sufficiently explicated. However, as illustrated above, Paul emphasizes that decision-making processes within our cultural paradigm are primarily governed by our pursuit of happiness. Furthermore, subjective values play a particular functional role in Paul's argument; (authentic) agents use subjective values to choose options that maximise their expected value. So, if we understand the notion of happiness very broadly within affective forecasting studies, this should be compatible with Paul’s view. The idea is as follows: When people are asked to rate their level of happiness in affective forecasting questionnaires, a higher happiness rating corresponds to a higher subjective value in that they both reflect a better outcome. Of course, in many affective forecasting studies participants are not asked about how an experience will contribute to their overall level of happiness. Instead, forecasters are sometimes asked about particular affective states. Obviously, it is not Paul's position that subjective values are the same as affective states. We have, therefore, excluded such studies from our meta-analysis and only include those that study happiness (see Sect. 5).

Admittedly, there is another way of criticising our operationalization. Paul argues that some experiences can have high intrinsic, revelatory subjective value, even if these experiences make the respective person less happy.^9^ The idea is that some experiences “have subjective value in virtue of what they teach us through the discovery of lived experience, a value that extends past their first-order qualitative character” (p. 92). Paul calls this value *revelatory*, and asserts “we might choose to have an experience because of its revelatory character, rather than choosing it because what it is like is in some way pleasurable or enjoyable” (p. 93). We will address this issue in Sect. 6.3.

It is also important to emphasize that we do not claim here that subjective values are reducible to hedonism (which Paul explicitly rejects). Our emphasis on subjective values does not mean we are committed to hedonism, such as sensory hedonism, which is probably the most well-known variety of hedonism. According to sensory hedonism, happiness is reducible to sensory pleasure, that is, it is reducible to pleasant sensations and pleasant experience. Again, the idea is that, whatever a subjective value is composed of, it is a measure of happiness and can be quantified on a one-dimensional scale. There is a subtle difference: While hedonism states that pleasantness is the only thing that is intrinsically valuable about experience, we remain agnostic on this issue. So, in contrast to Kauppinen (2015), for example, we do not argue that the intrinsic value of an experience is determined by its broad hedonic quality (an argument against Paul, 2014; Paul argues that the intrinsic value of an experience is also determined by some non-hedonic features, such as variety and novelty).^10^

To sum up, affective forecasting and happiness are promising operationalizations of cognitive modelling and subjective value. The reason is that affective forecasting studies measure how accurately people predict future happiness, whereby a higher happiness rating corresponds to a higher subjective value.

## Meta-analysis

### Method

The empirical claim of this essay relies on a meta-analysis conducted by Levine et al. (2012) on affective forecasting. For our analysis, we looked into each of the 84 studies listed in their meta-analysis to categorize them based on the criterion of whether the affective forecasting task was concerned with transformative or non-transformative experiences. Studies were categorized as investigating transformative experiences if the experience was epistemically and personally transformative. To rehearse, *epistemically transformative* means that the experience was new and of a different kind such that one would not know what it was like to have that experience unless one actually had it. *Personally transformative* means that the experience will influence the agent’s preferences.

Studies were categorized as investigating non-transformative experiences if these experiences were neither personally nor epistemically transformative. Probands probably had this kind of experience before and it did not change their personality in any important way.^11^

However, many of the studies in Levine et al.’s (2012) meta-analysis do not fit our criteria and have, therefore, been excluded; the excluded studies are listed in the appendix. The two most important reasons are:

1. Roughly 30 percent of studies did not ask participants about happiness in general. Instead, participants were asked to anticipate the intensity of emotions. Obviously, anticipating the intensity of particular emotions is neither the task of interest in this paper nor is it in line with our operationalization of cognitive modelling.
2. The effect size is not solely based on a transformative or non-transformative experience.

We compared the weighted means of the effect sizes (Hedges’s g) in the category of transformative and non-transformative experience. The effect sizes indicate the accuracy of probands’ predictions of their future happiness. A large effect size indicates that people strongly overestimate their reaction. A small effect size indicates that people only barely overestimate their reaction. A negative effect size indicates that people underestimate their reaction. It has to be noted, though, that we used absolute values for the analysis. The reason is that negative effect sizes would have had compensating effects (summing a large negative effect size and a large positive effect size would have led to a small effect size).

As a rule of thumb, an effect size between 0.2 and 0.5 is considered a small effect; an effect size between 0.5 and 0.8 is considered a medium effect; an effect size above 0.8 is considered a large effect (Lenhard & Lenhard, 2016).

The effect sizes calculated by Levine et al. (2012), the sample size of each study and whether the study concerns transformative experiences can be found in Table 1 in the “Appendix”.

The formula for the weighted average of various effect sizes of k studies is

1 $$d\left( w \right) \equiv w\left( 1 \right) \cdot d\left( 1 \right) + \cdots + w\left( k \right) \cdot d\left( k \right),$$

where w(1)…w(k) are non-negative weights that sum to unity and d(1)…d(k) are the individual effect sizes (Hedges & Olkin, 1985, p. 109). The weights can be approximated as follows:

2 $$w_{i} \cong \frac{{\tilde{n}_{i} }}{{\mathop \sum \nolimits_{j = 1}^{k} \tilde{n}_{j} }},$$

where $\tilde{n}_{i} = \frac{{\tilde{n}_{i}^{E} \cdot \tilde{n}_{i}^{C} }}{{\tilde{n}_{i}^{E} + \tilde{n}_{i}^{C} }}$ and n refers to the sample size (Hedges & Olkin, 1985, p. 110). The indexes E and C refer to the experimental and control groups. Calculated this way, the effect sizes of larger samples have more weight than those of smaller samples. This is because the estimates from larger studies are likely to be more precise. However, only a few affective forecasting studies include control groups, as there is no treatment that has to be tested.

One could object that it is possible that the mere act of forecasting will influence the experience. However, as most studies in the affective forecasting literature use between-subject designs, such expectation or contrast effects are negligible. Thus, we estimated each weight w_i_ by dividing the respective sample size n_i_ by the sum of all sample sizes. The weight calculated this way still accounts for the idea that effect sizes based on larger sample sizes are probably more precise and should therefore have more weight in calculating the overall average. Following Delacre et al. (2017), a two-tailed Welch’s *t*-test was used to compare the calculated means.

The null hypothesis was that the two groups (transformative experience forecasters and non-transformative experience forecasters) *do not* differ from each other. The alternative hypothesis was that the two groups *do* differ from each other. According to the null hypothesis, affective forecasts *would be* equally accurate. According to the alternative hypothesis, affective forecasts *would not be* equally accurate.

As the Welch’s test cannot establish whether two effect sizes are equal in a relevant sense, an equivalence test, the two one-sided test (TOST), was subsequently run (Lakens, 2017). The meaningfulness of equivalence tests heavily depends on a well justified equivalence margin, or smallest effect size of interest (SESOI; Lakens, 2017; Walker & Nowacki, 2010). However, no such SESOI has been established in the literature on the impact bias or transformative experiences. We therefore investigated the effect sizes considered clinically relevant in the psychological literature and opted for the smallest found, which is 0.24 (Cuijpers et al., 2014; see Steinert et al., 2017, for a review on reported cut offs for clinically irrelevant effects). This corresponds to a small effect size.

After exclusion criteria were applied, we included four studies investigating transformative experiences and 44 studies investigating non-transformative experiences. This amounts to 926 probands in the former category and 4104 probands in the latter category. The applied Welch’s *t*-test is the appropriate test for the resulting quantitative imbalance and inequality of variance.

### Result

The weighted average effect size of studies on non-transformative experiences is g = 0.6969. The weighted average effect size of studies on transformative experiences is g = 0.7057.

Welch’s *t*-test indicates that the effects do not significantly differ from each other, t(2367) = 0.6967, p = 0.4860. The TOST procedure based on Welch’s *t*-test reveals that the observed effect size was significantly within the equivalence bounds of d = − 0.24 and d = 0.24, t(2367) = 7.29, p < 0.001 (Lakens, 2017).

**Fig. 1:**
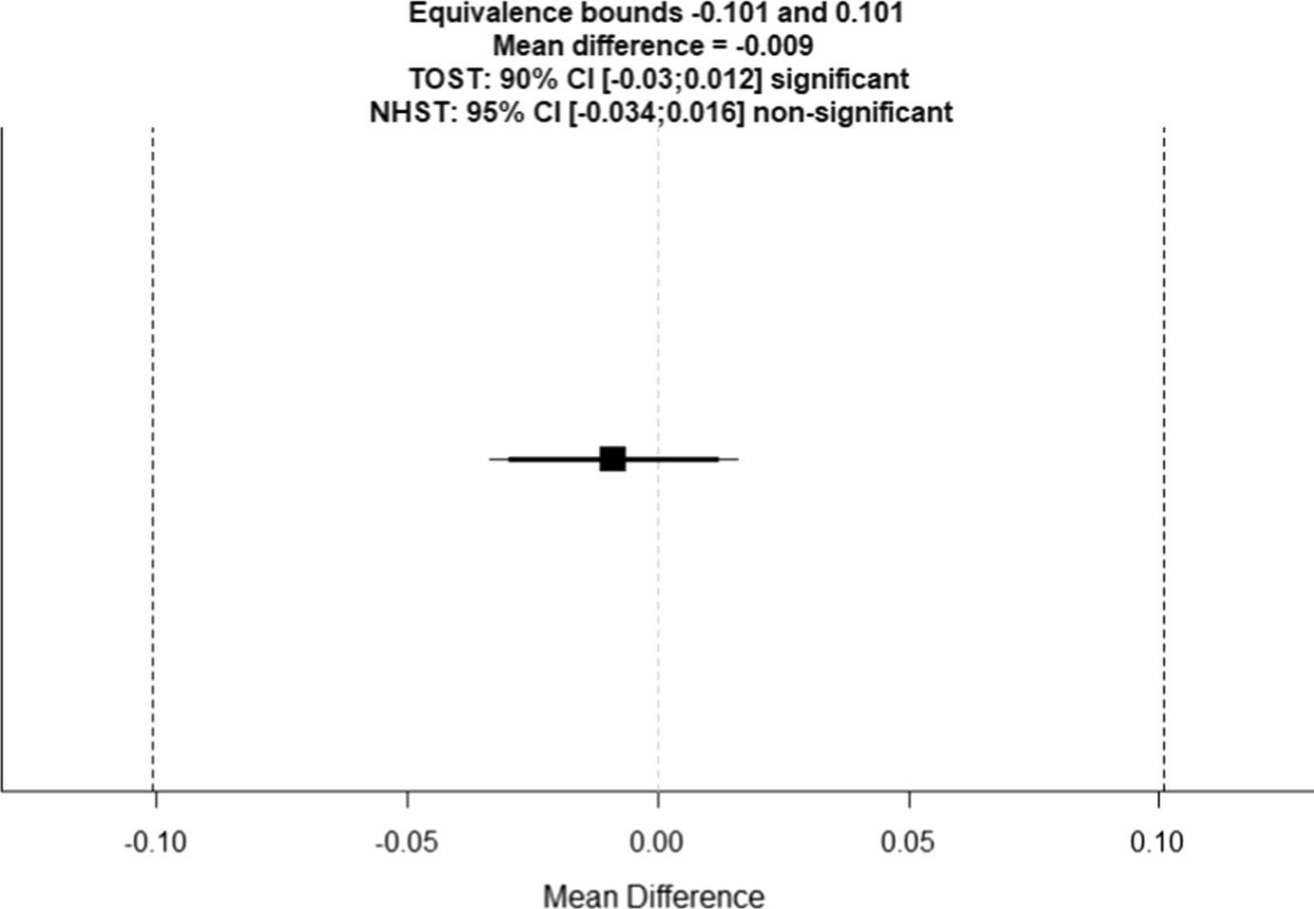
The graph shows in raw scale units the observed mean difference (− 0.009) as a black square, the equivalence bounds (− 0.101 and 0.101) as vertical dashed lines as well as the 90% confidence interval (CI) [− 0.03; 0.012] as a thick horizontal line and the 95% CI [− 0.034; 0.016] as a thin horizontal line. The raw values are derived from the values reported above but are independent of standard deviation. This allows the graph to be interpreted in a more intuitive way. The mean difference falls within the interval indicated by the equivalence bounds. Hence, the effect sizes are statistically equivalent

The null hypothesis is therefore not rejected and the equivalence test suggests statistical equivalence of the two effect sizes (see Fig. 1).

### Discussion

In our meta-analysis, we have analysed empirical evidence that decision-makers’ performances in affectively forecasting transformative experiences do not systematically differ from decision-makers’ performances in affectively forecasting non-transformative experiences. This is to say that people incorrectly predict the *intensity* and *duration* of their future happiness independently of whether the experience is transformative. In virtue of our operationalization this means that transformative and non-transformative experiences are equally problematic with respect to the ability to cognitively model. If the transformative nature of transformative experiences *caused* problems with cognitive modelling, we should have found an effect in our meta-analysis. However, we did not find such an effect. Hence, our study falsifies Paul’s main argument that cognitive modelling fails because of the transformative nature of the decision.

## Objections

In the previous section, we presented the result of our meta-analysis, which indicates that people incorrectly predict their future level of happiness independent of whether the experience is transformative. In the remainder of the paper, we address four objections against our approach and results. The first concern is that cognitive modelling is connected to a particular concept of happiness not addressed by the affective forecasting literature. The second worry claims that we do not know whether affective forecasting is done from a first-person or third-person perspective. The third challenge asserts that subjective value and happiness come apart when basing one’s choice on the revelatory value. The fourth objection points out that the status of the impact bias is controversial and contends that the meta-analysis is therefore questionable.

While the first three objections relate to the proposed operationalization of cognitive modelling, the fourth objection states that we should not transfer controversial issues on impact bias over to the research on transformative experiences.

### Happiness and Affective Forecasting

One might doubt our interpretation of Paul’s view that subjective values and happiness can both be understood in broad ways within her framework. Instead, we should rather rely on a specific concept of happiness.

The concept ‘happiness’ has two entirely distinct meanings. On the one hand, some philosophers use the word ‘happiness’ as a value term, more or less synonymously with ‘well-being’ or ‘flourishing’ (or ‘eudaimonia’). When these philosophers ask, ‘What is happiness?’ they are asking questions such as: What is good for a person? What should one do in order to be better off? On the other hand, some philosophers use the concept ‘happiness’ not as a value term, but simply as a descriptive psychological term. In other words, these philosophers use ‘happiness’ to denote a mental state. Thus, when they ask, ‘What is happiness?’, the question is: What is the mental state that we call ‘happiness’? Importantly, there are three main philosophical approaches concerning what mental state happiness is: hedonism, the life satisfaction theory and the emotional state view. Simply put, according to hedonism, happiness is identical to pleasure and the absence of pain. The life satisfaction theory states that happiness is identical to having a favourable attitude towards one's life as a whole. And, according to the emotional state view, happiness is identical to a positive global emotional condition.

Obviously, such essential conceptual differentiations as those mentioned above are not captured if psychologists in the affective forecasting literature ask questions like ‘How happy would you say that you are these days?’ We cannot rule out that, according to Paul, affective forecasting and cognitive modelling relate to different concepts of happiness. The reason is that we do not know Paul's exact view on the relationship between happiness, cognitive modelling and subjective values. However, affective forecasting questionnaires do not rely on one concept of happiness more than another. So, if Paul agrees to our conjecture that subjective values reflect a happiness ranking of some sort, there is no reason to believe that affective forecasting questionnaires aim for another concept of happiness.

### First Versus Third Personal Perspective and Affective Forecasting

It seems that there are at least two ways of acquiring ‘what it is like’-knowledge (see Cath, 2019, for a detailed discussion). One possibility is to acquire it from a first-person perspective (e.g. by using cognitive modelling). Another possibility is to acquire it from a third-person perspective (e.g. by consulting testimony).

The third objection goes along the lines of this:

1. Our finding—that affective forecasting is equally accurate for transformative and non-transformative experiences—only challenges Paul's view if affective forecasting is done from a first-personal perspective.
2. Affective forecasting, however, is done from a third-personal perspective.

Consequently, the results of the meta-analysis would only suggest that third-personal estimates of value are equally accurate for transformative and non-transformative experiences. Even though this might be seen as interesting finding in itself, it does not stand in contradiction to L.A. Paul’s claim.

Is this objection plausible? We think that (1) is correct. Affective forecasting is not an appropriate operationalization of cognitive modelling if it is exclusively done from a third-person perspective. Admittedly, we cannot exclude this possibility, because affective forecasting studies do not consider whether participants are forecasting from a first-person or third-person perspective. Note, however, that Paul often emphasizes that the first-person perspective is central for anticipating one’s future (see, e.g. Paul, 2014, pp. 42–43). The claim that affective forecasting is exclusively done from a third-person perspective does not seem to be compatible with Paul’s position that the decision outcomes are described as ‘what it is like locutions’ determined by cognitive modelling (p. 26). Moreover, and more importantly, it is quite conceivable that forecasters answer general questions of happiness such as, ‘How will *you* feel?’ from a first-person perspective (we have ruled out studies about particular emotions). So, we don’t believe that (2) is correct and we therefore regard the objection as unconvincing. But we grant that we lack the data to safely rule (2) out.

### Revelatory Approach and Affective Forecasting

L.A. Paul proposes the *revelatory approach* wherein an agent should base transformative decisions on the so-called *revelatory value* (for a discussion, see, e.g., Kauppinen, 2015; Shupe, 2016). The basic idea is that instead of basing a transformative choice on a prediction regarding one’s future subjective value, one should make such a decision by asking oneself how one values new experiences and new selves. For example, if you deliberate about personal transformative choices, such as being a parent for the first time, the crucial question would be how much you value discovering what it is like to be a parent or become a different self (Paul calls this the revelatory value).

Based on the revelatory approach, the third objection is that some experiences can have high revelatory value, even if they make the agent less happy. In these instances, it would not matter if one could not assign a subjective value to the experience of, say, parenthood, as long as one could assign a subjective (revelatory) value to the outcome of *discovering* what it will be like to become a parent. In such cases, where the what it is like character of the experience is not the dominant factor, subjective value and happiness can come apart.

Are we therefore mistaken in operationalizing subjective value as happiness, as argued in Sect. 4.3? We think not. The revelatory approach is Paul’s proposed solution to the problems that arise due to the failure of cognitively modelling what transformative experiences will be like (Paul, 2014, p. 120). In contrast, this paper has investigated whether the transformative nature of transformative experience is problematic for the ability to cognitively model in the first place. When people are asked in affective forecasting questionnaires to predict how happy they will be after having had a certain experience, they are being asked to run a cognitive simulation of *what it will be like* to have that experience. When they run this simulation, we, and Paul—as we interpret her—think that it is plausible that people care about their happiness or well-being.

Thus, our research question is not about the revelatory approach or revelatory forecasting (as far as we know, there are no studies on revelatory forecasting). It is possible that future research will show that forecasts of revelatory value are differently accurate for transformative and non-transformative experiences. The revelatory value, however, seems to be determined independent of cognitive modelling in Paul’s framework. In order to establish the revelatory approach, Paul reformulates the structure of the decision problem such that the outcomes do not involve experiencing but rather discovering the transformative outcome. One could say that Paul reformulates the structure of the decision problem in a way that it does not involve cognitive modelling anymore. If you deliberate about trying a durian fruit for the first time, for instance, the relevant outcomes are *discovering the taste of durian* and *avoid discovering the taste of durian*. The point is that these outcomes are determined independently of the experienced taste and, thus, do not seem to require cognitive modelling. Put differently, one uses cognitive modeling to find out what an experience X is like but one does not use cognitive modeling to find out whether one wants to discover X or to avoid discovering X.

### Impact Bias and Meta-analysis

The status of the impact bias is debated. For instance, Levine et al. (2012) argue that a large part of discrepancies between predictions and experiences recorded in the affective forecasting literature is due to a procedural artefact common to most affective forecasting questionnaires. According to Levine et al. (2012), people can accurately predict their emotional reaction *towards events*; an artifact of the studies contributes to people’s tendency to overestimate the intensity of their general feelings.

The present paper does not defend the claim that the impact bias exists (for such a discussion, see, e.g. Wilson & Gilbert, 2013; Levine et al., 2013). Our meta-analysis includes the studies from which Levine et al. (2012) hypothesized that a procedural artifact increases the impact bias (and those studies without this artefact).

Are we therefore mistaken in basing our criticism of Paul’s misconception about cognitive modelling on affective forecasting studies? No! The core of our paper, i.e. the empirical result of the categorized meta-analysis, does not presuppose anything about the impact bias. The impact bias is the most commonly used measure for affective forecasting accuracy; whether it is a phenomenon of significance is irrelevant to our main hypothesis. The only deciding question is: Are there systematic differences in forecasting accuracy between transformative and non-transformative experiences? This question can conveniently be investigated by analysing the literature on the impact bias without accepting any presupposition about the impact bias itself.

## Conclusion

L. A. Paul's book, *Transformative Experience*, has been one of the most widely discussed works in analytic philosophy in the last few years. Paul's claims are striking, but appeals to intuition do a lot of the work. Until now, we knew very little about the connection between cognitive modelling and transformative experiences. There has been a lack of empirical analyses in this area. In particular, we did not yet know if Paul is correct in claiming that transformative experiences pose special problems for the ability of cognitive modelling. This paper provides the first meta-analysis that investigates whether cognitive modelling fails because of the transformative nature of the experience. In order to do that, we have operationalized the ability to cognitively model as the capacity of affective forecasting, and compared transformative and non-transformative experiences with respect to the ability of affective forecasting. According to our results, it is *not* the transformative nature of transformative experiences that creates the problems with affective forecasting. Thus, *if* we have correctly operationalized the involved concepts, Paul’s main argument is mistaken.

## Appendix

See Table 1.

**Table 1:** Categorized meta-analysis

Study name	Study	Sample size	Effect size (Hedges’s g)	TE or nonTE
Anrade, and Van Booven (2010)	Study 1	43	− 0.45	nonTE
	Study 2	61	− 0.32	nonTE
Ayton et al. (2007)	Study 1	114	0.36	nonTE
Carlsmith et al. (2008)	Study 1	48	1.14	nonTE
	Study 3	75	0.83	nonTE
Dunn, and Ashton-James (2008)	Study 1	161	1.37	nonTE
	Study 2	173	0.60	nonTE
	Study 3	302	1.16	nonTE
Dunn et al. (2007)	Study 1	62	0.03	nonTE
	Study 2a	76	− 0.25	nonTE
	Study 2b	46	− 0.31	nonTE
	Study 3	84	0.70	nonTE
	Study 4	87	0.50	nonTE
Dunn et al. (2003)	Study 1	84	1.01	nonTE
	Study 2	234	1.00	nonTE
Emanuel et al. (2010)	Study 1	220	1.36	nonTE
Finkenauer et al. (2007)	Study 1	37	0.87	nonTE
Gilbert et al. (1998)	Study 2	220	0.69	TE
	Study 3	57	0.67	nonTE
	Study 4	73	0.46	nonTE
	Study 6	91	1.06	nonTE
Greitemeyer (2009)	Study 1	55	1.21	nonTE
	Study 2	94	0.59	nonTE
	Study 3	129	0.68	nonTE
Hoerger et al. (2009)	Study 1	180	0.84	nonTE
Hoerger et al. (2010)	Study 1	57	0.34	nonTE
Hsee, and Zhang (2004)	Study 1	249	0	nonTE
	Study 3	243	− 0.36	nonTE
Kermer et al. (2006)	Study 1	54	0.77	nonTE
Kurtz et al. (2007)	Study 1	42	0.45	nonTE
Lam et al. (2005)	Study 1	109	0.66	nonTE
	Study 2	40	0.56	nonTE
Meyvis et al. (2010)	Study 1	19	0.94	nonTE
	Study 2	73	0.79	nonTE
	Study 4	40	0.68	nonTE
Morewedge et al. (2007)	Study 2, 4	221	− 0.27	nonTE
	Study 6	72	0.52	nonTE
Riis et al. (2005)	Study 1	98	0.15	TE
Sevdalis et al. (2009)	Study 1	20	0.32	nonTE
	Study 2	22	0.36	nonTE
	Study 3	46	− 0.58	nonTE
Sieff et al. (1999)	Study 1	91	0.58	TE
Smith et al. (2008)	Study 1	517	0.84	TE
Wilson et al. (2003)	Study 1	52	0.88	nonTE
Wilson et al. (2000)	Study 1,2	132	0.71	nonTE
	Study 3	27	1.1	nonTE

## Excluded Studies

Buehler and McFarland (2001), Crawford et al. (2002), Fernandez-Duque and Landers (2008), Gilbert et al. (2004), Gilbert et al. (1998), study 1, study 5, Hartnett and Skowronski (2010), Hoerger and Quirk (2010), Kawakami et al. (2009), Kermer et al. (2006), study 2, Koo et al. (2008), Ku (2008), Mallett et al. (2008), Nielsen et al. (2008), Sevdalis and Harvey (2007), Tomlinson et al. (2010), Van Dijk (2009), Van Dijk et al. (2008), Wilson et al. (2005) and Wirtz et al. (2003).

## Footnotes

1 For a sophisticated discussion on Paul’s claim that if one aims to be authentic, transformative decisions cannot be made rationally, see, e.g., Friedman (unpublished manuscript), Barnes (2015), Campbell (2015), Sharadin (2015), Dougherty et al. (2015), Kauppinen (2015), McKinnon (2015), Bykvist and Stefánsson (2017), Reuter and Messerli (2018) and Pettigrew (2015, 2016, 2020).

2 Strictly speaking, if an experience is only personally transformative the decision maker could generate subjective values based on what it would be like for her current self. A complete ranking, however, would have to involve the subjective values of her future self.

3 We would like to thank an anonymous reviewer for this journal for very helpful comments on this issue.

4 One might object that Paul rather has a relation in mind that can be described by the logical operator implication. For if A (the transformative nature) implies B (problems with cognitive modelling), non-A does not imply non-B. This, however, would not be a plausible interpretation of Paul’s position. She makes clear that transformative experiences are a phenomenon worth acknowledging in virtue of the problems that are caused by their transformative nature. The emphasis in Paul’s book is not simply on the existence of transformative experiences, but on them being problematic for rational choice theory. Accordingly, the problems created by the transformative nature are exactly what differentiates transformative from non-transformative experiences. A more accurate representation of her claim, then, is that the problems with cognitive modelling appear only if the respective experience is transformative.

5 We are grateful to an anonymous reviewer for this journal for very helpful comments on this section.

6 One might argue that there is another candidate concept that could avoid the two problems discussed above (i.e., accuracy and connection to happiness): so-called *episodic prediction* (Szpunar et al., 2014). The aim of Szpunar and colleagues’ paper is to organize the research on prospection by introducing a respective taxonomy. They define episodic prediction as “the estimation of […] one’s reaction to a specific autobiographical future event” (Szpunar et al., 2014, p. 18,417), whereby the process of episodic prediction work as follows: “people base their predictions on episodic simulations of the future” (p. 18,417). This fits perfectly with how we interpret L.A Paul’s account of cognitive modelling: One runs a cognitive simulation to project oneself into experiencing each outcome and based on that simulation one predicts how one will feel in the respective situation. Interestingly, Szpunar et al. themselves suggest that the clearest way of investigating episodic prediction is shown in the literature on affective forecasting.

7 We are grateful to an anonymous reviewer for this journal for very helpful comments on this section.

8 Another example in which the association between happiness and subjective values is apparent is this: So, in this scenario, when you deliberate about whether to have your first child, to do it rationally, you are supposed to (at least approximately) assess your subjective values for the outcomes stemming from choosing to have a child or remain childless, determine any relevant probabilities, identify the act that maximizes your expected subjective value, and then act in accordance with your preferences, that is, choose the act with the highest expected subjective value. (pp. 75). In this quote it becomes clear that to act in accordance with your preferences means choosing the act with the highest subjective value. It follows that if one prefers action A over action B, one expects that action A will, *ceteris paribus*, have a higher subjective value.

9 We are grateful to an anonymous reviewer for this journal for very helpful comments on this section.

10 Note, however, that there is support for Kauppinen's position from cognitive psychology, as regards the claims that (a) all experiences share a hedonic quality (see e.g. Kahneman 1999) and that (b) identifying collative variables, such as variety and novelty, demands cognitive processing, but the evaluation of a stimulus is often made affectively and not cognitively (see, e.g. Zajonc 1980).

11 The experiences coded as *transformative* include receiving tenure, receiving an HIV diagnosis, having to live with several serious illnesses that require hemodialysis and having experienced a kidney transplant. One could question the connection of these experiences to paradigmatic cases of transformative experiences from the literature, such as having a child or becoming a vampire. We grant that these are cases that have not been in the spotlight of debate on transformative experiences. However, they do formally align with the criteria for transformative experiences. Furthermore, we think that these cases *should* be more relevant in the transformative experiences literature since they concern substantial subjects of modern life: realizing or failing to realize one’s career goals and having to deal with significant health issues.
